# Probing the power grid of C_4_ plants: how the cyclic electron transport fuels and protects photosynthesis

**DOI:** 10.1093/plphys/kiag179

**Published:** 2026-04-03

**Authors:** Matheus E Bianconi

**Affiliations:** Assistant Features Editor, Plant Physiology, American Society of Plant Biologists; Laboratoire de Recherche en Sciences Végétales (LRSV), Université de Toulouse, CNRS, UPS, Toulouse INP, Castanet-Tolosan, France

Light that reaches chloroplasts drives the production of ATP and NADPH, which are required for CO_2_ fixation during photosynthesis. Such light-dependent reactions occur in the system of membranes embedded in chloroplasts known as thylakoids. Thylakoid membranes harbor two types of light-harvesting protein complexes, namely photosystems (PS) I and II. In PSII, light energy drives the oxidation of water, releasing electrons that pass through a series of redox reactions via a protein complex known as cytochrome *b*_6_*f*, and PSI, ultimately reducing NADP^+^ into NADPH. This pathway, known as linear electron transport (LET), also generates a proton motive force (*pmf*) in the lumen of thylakoids that fuels ATP production via ATP synthase activity (Fig. 1). The redox reactions in thylakoids therefore work as a power transmission line that has to be tightly regulated not only to match the energetic demand of carbon fixation reactions of photosynthesis, but also to avoid overload when this demand decreases, for example when stomata close and photosynthesis is limited by CO_2_ influx. Such downregulation is fundamental in preventing the formation of reactive oxygen species (ROS), which can damage the photosynthetic apparatus, reducing the photosynthetic rate and growth (Krause 1988).

**Figure 1:**
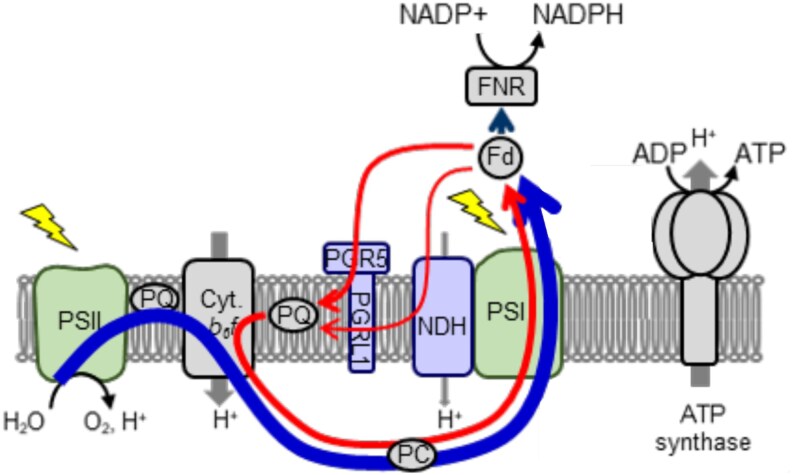
Electron transport pathways in thylakoid membranes. Blue and red lines indicate linear electron transport (LET) and cyclic electron transport around photosystem I (CET-PSI). Selected components of thylakoid membrane are indicated: photosystem II (PSII), plastoquinone (PQ), plastocyanin (PC), ferredoxin (Fd), ferredoxin-NADP^+^ reductase (FNR), PROTON-GRADIENT REGULATION 5 (PGR5)/PGR5-like photosynthetic phenotype 1 (PGRL1), chloroplast NADH dehydrogenase-like (NDH). Modified from Nakamura et al. (this issue). Schematic representation of a thylakoid membrane showing components of the electron transport flow.

Thylakoid membranes also support an alternative energetic pathway known as the cyclic electron transport around PSI (CET-PSI; Arnon et al. 1967). In this route, the electron that flows to PSI is redirected to cytochrome *b*_6_*f*, which addresses it back to PSI, releasing protons in the thylakoid lumen during this process and contributing to ATP formation that is decoupled from NADPH production (Fig. 1). The CET-PSI is particularly important for C_4_ plants, which have higher ATP demand than C_3_ plants (Yin and Struik 2018). In C_4_ leaves, CO_2_ is initially fixed in mesophyll cells (MC) into a C_4_ acid that is shuttled to the adjacent bundle sheath cells (BSC), where it is decarboxylated, releasing CO_2_ that is fixed by Rubisco. The extra ATP required by C_4_ plants is used for the regeneration of the initial CO_2_ acceptor in the MC. In some C_4_ species, NADP-malic enzyme (NADP-ME) is used for decarboxylating the C_4_ acid in the BSC, and this forms NADPH as a byproduct. This additional source of NADPH translates into a smaller LET demand in the BSC, and indeed some C_4_ NADP-ME species have agranal chloroplasts in the BSC, with reduced PSII and LET activity (Woo et al. 1970). The differential control of light-dependent reactions in MC and BSC chloroplasts has recently regained attention due to its relevance for understanding how the C_4_ machinery evolved (Wang et al. 2026) and for C_4_ engineering efforts (Ermakova et al. 2020). In this issue of *Plant Physiology*, Nakamura and colleagues (2026) provide new mechanistic insights into how the CET-PSI pathway contributes to photosynthesis and photoprotection in C_4_ plants using the model plant *Flaveria bidentis*.

The CET-PSI has two known routes for passing electrons to cytochrome *b*_6_*f*. One depends on PROTON-GRADIENT REGULATION 5 (PGR5)/PGR5-like photosynthetic phenotype 1 (PGRL1), and a second relies on the chloroplast NADH dehydrogenase-like (NDH) complex. During CET-PSI, an electron is transferred from PSI to ferredoxin (Fd), which then passes it to plastoquinone (PQ) either via PGR5/PGRL1 or NDH (Fig. 1). PQ then reduces plastocyanin (PC) via cytochrome *b*_6_*f*, releasing two (PGR5/PGRL1 pathway) or four (NDH pathway) protons in the thylakoid lumen, while PC restarts the CET by reducing Fd via PSI. The specific roles of PGR5/PGRL1 and NDH have been studied in C_3_ and C_4_ species. In C_3_ plants, the PGR5/PGRL1-dependent pathway contributes mainly to photoprotection by preventing PSI overreduction (Suorsa et al. 2012), while in some C_4_ plants the NDH-dependent route mainly contributes to ATP formation (Takabayashi et al. 2005). However, the mechanism by which CET-PSI protects C_4_ leaves from photoinhibition, and the specific role of PGR5/PGRL1 and NDH in MC and BSC remained unclear.

Nakamura et al. address these gaps using RNAi knockdown (KD) lines of *F. bidentis*. By crossing PGR5/PGRL1- and NDH-RNAi KD lines together, they generated double KD offspring with drastically impaired CET-PSI, and conducted growth, physiological, and biochemical analyses to determine the contribution of each CET-PSI route to photosynthesis and photoprotection. They report severe growth reduction in the double KD line as a result of reduced net CO_2_ assimilation and electron transport rates, which were lower than both the single KD lines and the wild type. To elucidate the mechanistic link between CET-PSI and impaired photosynthesis, the authors performed detailed assays probing chlorophyll fluorescence in isolated thylakoids. Among their results is the finding that the reaction center of PSI (P700) is over-reduced in the KD lines, which limits the capacity of PSI to accept electrons from PC, therefore impairing the capacity of photochemical energy dissipation. To test the hypothesis that CET-PSI impaired lines would be particularly sensitive to high light, they grew plants at low light levels (80–100 μmol m^−2^ s^−1^) and assessed the amount of oxidized P700 before and after exposing the plants to a 15-min pulse of saturating light (2,200 μmol m^−2^ s^−1^) followed by dark acclimation. They observed that, while wild-type plants could recover normal levels of oxidized P700 after dark acclimation, CET-PSI deficient lines could not, with double KD lines recovering only 30% of pre-exposure levels, which are lower values than either parental KD line. Their results support the idea that both CET-PSI routes are necessary not only for energy production in C_4_ plants, but also for photoprotection, through preventing overreduction of thylakoid membrane components when light energy input surpasses the photosynthetic demand.

Nakamura et al. provide a detailed mechanistic explanation for the regulatory mechanisms underlying the notable efficiency of the photosynthetic apparatus of C_4_ plants. Some questions, however, deserve further investigation. Agranal BSC chloroplasts as those in *F. bidentis* were also observed in other NADP-ME species, but they are not the rule in C_4_ plants (Ueno et al. 2005). Determining if agranal BSC results from lineage-specific contingencies or adaptation to particular environments would help us better understand how cell energetic functions are partitioned between MC and BSC during C_4_ evolution. This could in turn have important implications for creating optimized designs of C_4_-engineered crops.

## Recent research articles in *Plant Physiology:*

Peltier et al. (2024) investigate the contribution of alternative electron transport pathways to energy production required for CO_2_ fixation in the model microalgae *Chlamydomonas reinhardtii*, providing a perspective of the importance of the cyclic electron transport in the context of another CO_2_-concentrating mechanism.

Lauterbach et al. (2025) provide insights into how the C_4_ machinery evolved from a C_3_ ancestral by conducting a detailed comparative study of leaf transcriptomes in closely related C_3_, C_3_-C_4_ intermediate, and C_4_ species of the grass genus *Neurachne*.

## Data Availability

No new data were generated or analysed in support of this News and Views article.
